# Relationship between Serum Methadone Concentration and Cold Pressor Pain Sensitivity in Patients Undergoing Methadone Maintenance Therapy

**Published:** 2018

**Authors:** Zalina Zahari, Chee Siong Lee, Muslih Abdulkarim Ibrahim, Nurfadhlina Musa, Mohd Azhar Mohd Yasin, Yeong Yeh Lee, Soo Choon Tan, Nasir Mohamad, Rusli Ismail

**Affiliations:** a *Faculty of Pharmacy, Universiti Sultan Zainal Abidin (UniSZA), Besut Campus, Terengganu, Malaysia. *; b *Pharmacogenetics and Novel Therapeutics Cluster, Institute for Research in Molecular Medicine (INFORMM), Universiti Sains Malaysia (USM), Kelantan, Malaysia. *; c *Emergency and Trauma Department, Hospital Sultanah Aminah, Johor, Malaysia. *; d *Department of Pharmacology and Toxicology, College of Pharmacy, Hawler Medical University, Hawler, Iraq. *; e *Department of Psychiatry, School of Medical Sciences, USM, Kelantan, Malaysia.*; f *School of Medical Sciences, Universiti Sains Malaysia, Kelantan, Malaysia. *; g *Faculty of Medicine, Universiti Sultan Zainal Abidin (UniSZA), Medical Campus, Terengganu, Malaysia.*

**Keywords:** Cold pressor pain, Pain threshold, Pain tolerance, Pain intensity, Methadone, Methadone maintenance therapy (MMT)

## Abstract

Hyperalgesia is a common clinical phenomenon among opioid dependent patients on methadone maintenance therapy (MMT) and it may be associated with undertreated pain and/or therapeutic failure. This study aimed to investigate association between serum methadone concentration (SMC) and cold pressor pain responses. Cold pressor pain responses in 147 opioid dependent patients on MMT were assessed using cold pressor test (CPT) at 0 h and at 2, 4, 8, 12, and 24 h after the dose intake. Blood samples were collected at 24 h after the dose. Serum methadone concentrations were measured using the Methadone ELISA kit and classified into two categories: < 400 ng/mL and ≥ 400 ng/mL. Eighty-eight patients (59.9%) had trough concentrations of < 400 ng/mL and 40.1% had trough concentrations of ≥ 400 ng/mL. There were significant effects of SMC on the cold pressor pain threshold (*p* = 0.019). Patients with concentrations < 400 ng/mL had significantly higher (almost 60% higher) cold pressor pain threshold (adjusted mean (95% CI) = 30.15 (24.29, 36.01) s) compared to those with concentrations of ≥ 400 ng/mL (18.93 (11.77, 26.08) seconds). There was also a 20% difference in pain tolerance, and 6% difference in cold pressor pain intensity score, neither of which were significant statistically (*p* > 0.05). Our results suggest an association of trough methadone concentration with the cold pressor pain threshold among opioid dependent patients on MMT. It would be useful to study the mechanisms underlying this association to help managing pain in such a population.

## Introduction

Research evaluating the healthcare costs associated with treatment of opioid dependence with medications found that patients who received medications including methadone had lower hospital utilization and total costs than patients who did not receive them (1). However, it has been shown that patients on maintenance methadone treatment often receive under-treatment for pain. Reasons suggested were a lack of awareness among physicians about treatment of chronic and acute pain in this patient population. Methadone-maintained patients may receive inadequate doses of opioid analgesic for their pain (2-5). Poor pain management may be a risk factor for relapse for individuals with addiction in remission (6,7) and may contribute to discontinuation of methadone maintenance therapy (MMT). The consequent continued use of illicit opiates poses challenges in the treatment of patients with opiate dependence (5).

In addition to medical provider barriers, patient factors may also contribute to poor pain management in opioid dependent patients. Physiological and psychological aspects of active opioid addiction can make pain management more difficult (7). Opioid dependent patients frequently report increased pain sensitivity (8-16). Hyperalgesia, or oversensitivity to noxious stimuli, caused by chronic use of opioid drugs among patients on methadone therapy may be due to reorganization of nociceptive pathways in the brain (7, 9). Cross-tolerance to opioids may also be present (8, 12), and they may need more analgesia and it has been shown that opioid dependent patients require higher than normal doses of opioid analgesics. Despite significantly greater plasma morphine concentrations, methadone patients experienced minimal anti-nociception in comparison with controls (12). Although even higher morphine doses may achieve some pain relief, this may be at the cost of unacceptable respiratory depression (8). It is thus important for clinicians to understand pain sensitivity among patients on MMT for more effective pain management in this population.

Several studies on pain sensitivity among opioid dependent patients on MMT have been undertaken previously in several populations in California, USA (9-11,17,18), in Israel (16,19) and in Australia (8,12-15). 

Because of the hyperalgesia among opioid dependent patients on MMT may be associated with the under-treated pain and/or therapeutic failure (7), it is of major important that doctors or other health care providers working in maintenance programs to identify which patients might have increase susceptibility to methadone-induced adverse effects such as hyperalgesia.

Thus, assessing the relationship of methadone concentrations in blood with the corresponding pain sensitivity can be useful to improve pain management among opioid dependent patients on MMT. The present study investigated pain sensitivity by using cold pressor test (CPT) with methadone steady-state trough concentrations measured in a group of 147 opioid dependent patients on MMT, against a hypothesis that there are significant relationships between methadone concentration and pain sensitivity. 

## Experimental

*Patients*

Patients were consecutively recruited from those attending the Psychiatric Clinic, Hospital Universiti Sains Malaysia (HUSM), Psychiatric Clinic, Hospital Raja Perempuan Zainab II and eight other government MMT clinics in Kelantan. Opioid dependent patients on the MMT programme at a non-governmental organisation (NGO), SAHABAT between March and October 2013 were also asked to participate in this study. 

Inclusion criteria were: 

1. Malay for at least up to three generations.

2. Male aged more than 18 years.

3. Free of acute medical, surgical and psychiatric illness.

4. Free of acute or chronic medical, surgical and psychiatric illness that requires concurrent medical, surgical or psychiatric therapy.

5. Free of regular use of alcohol.

6. Free of intoxication.

7. Able to understand study protocols and to follow simple study instructions.

8. Willing to sign written informed consent.

Exclusion criteria were patients with or taking: 

1. diabetes mellitus. 

2. human immunodeficiency virus (HIV) and were on highly active antiretroviral therapy (HAART). 

3. major psychiatric illness such as schizophrenia.

4. taking illicit benzodiazepines, cannabinoids and barbiturates. 

5. peripheral vascular disease. 

6. regular anticonvulsants, neuroleptics or analgesics. 

7. chronic or ongoing acute pain. 

8. a history of analgesics ingestion within three days before the cold pressor test (CPT).

9. severe cognitive impairment which may interfere with pain assessments and/or communication.

Urine drug screens were performed twice in one week prior to enrollment to ensure that exclusion criteria were not met. The selected subjects were interviewed by the researcher based on a standard performa Information recorded included socio-demographic variables and other relevant information. 

The study was approved by the Human Research Ethics Committee (HREC), Universiti Sains Malaysia (USM) in Kelantan, Malaysia (Reference number: USMKK/PPP/JEPeM (253.3 [14]) and the Medical Research & Ethics Committee (MREC) at the Ministry of Health (MOH), Malaysia (Reference number: NMRR-13-524-16614).

*Cold pressor test (CPT)*

Pain sensitivity was assessed using the cold pressor test (CPT). The CPT method utilized in the current study was based on previous reports from Chen *et al.* (20) and Compton *et al*. (11). The reliability and validity of the CPT has also been extensively established (20-22). The CPT has been previously used extensively worldwide to characterize pain sensitivity among opioid dependent patients (8-10,12-16,19,23). 

The CPT has been considered the best pain induction technique to investigate pain sensitivity among methadone maintained patients as compared to the use of other pain induction techniques such as electrical stimulation (ES) (13,15,24).

The CPT apparatus consisted of a 48 quart cool box filled with a mixture of two-thirds crushed ice and one-third tap water. The resulting ice-water mix was stirred to maintain a constant temperature of 0 – 2 °C by adding ice with temperature constantly being monitored by a digital indoor-outdoor-thermometer (TFA Dostmann GmbH & Co.KG, Wertheim).

The pain threshold was defined as the mildest experience of pain that can be identified by a subject (i.e. time elapsed when the subject started to perceive pain after the immersion of hand). The pain tolerance was defined as the most severe pain that a subject was willing to tolerate (i.e. the time required for hand withdrawal). Both pain threshold and tolerance were quantified in sec. The CPT was truncated at 300 s, as after this time, numbness set in and pain diminished (11,25,26). Pain tolerance for subjects that did not withdraw their hand for the entire 300 s was recorded as 300 s. 

After withdrawal of the immersed hand, each subject was given a piece of dry towel to dry their hand. Immediately after hand withdrawal, subjects were asked to subjectively score their maximal pain intensity using a valid and reliable instrument, the 0-100 visual analogue scale (VAS), where zero (0) represented no pain and a hundred (100) represented the worst pain imaginable (27,28). 

Subjects were tested at 0 h [i.e. approximately 30 min before taking their morning dose of methadone (at about 8.00 AM)], and at 2, 4, 8, 12, and 24 h after the dose intake. We examined cold pressor responses six times over a 24 h period, in order to minimize the possible diurnal variation in cold pressor pain response (29). The test was administered by one trained research assistant (SHH).

*Blood sampling and methadone assay procedure*

Methadone steady-state trough concentration (30) was assessed assuming that methadone blood level at steady state reflects the receptor levels of methadone at the site of its action in the brain which are considered as necessary concentration in exerting the clinical effects 

(31-33).

Blood samples had been collected at 24 h after the dose intake [i.e. immediately (approximately 30 min) before taking their morning dose of methadone] from the branula inserted. 

Blood samples were filled in a labelled plain glass tube. The blood samples were allowed to clot by leaving it undisturbed at room temperature for 15 to 30 min. The tubes were kept in ice pack and were sent to the Pharmacogenomic laboratory at the Institute for Research in Molecular Medicine (INFORMM), USM, Kota Bharu, Kelantan.

**Table 1:** Demographic characteristics for the opioid dependent pwatients with a methadone concentration (at 24 h) of below 400 ng/mL and 400 ng/mL and above

**Variable**	**Total (** **N** **= 147)**		**< 400 ng/mL ** * ** (** **N** ** = 88)**		**≥ 400 ng/mL ** * **(** **N** ** = 59)**		**Mean difference** **(95% CI)**	***t*** **-statistic (** ***df*** **)**	***p*** **-value ** a
	**Mean**	**SD**	**Mean**	**SD**	**Mean**	**SD**			
Age (years)	36.86	6.13	36.75	6.13	37.10	6.19	-0.35 (-2.40, 1.70)	-0.34 (145)	0.735
Weight (Kg)	61.73	10.54	60.86	11.45	62.92	9.05	-2.05 (-5.56, 1.46)	-1.16 (145)	0.250
BMI (Kg/m^2^)	22.17	3.57	21.98	3.89	22.43	3.06	-0.46 (-1.65, 0.74)	-0.76 (145)	0.451
Methadone dose (mg)	72.70	28.25	67.10	27.61	81.02	27.59	-13.91 (-23.09, -4.73)	-3.00 (145)	0.003
Global PSQI score	5.32	2.71	5.24	2.97	5.37	2.25	-0.13 (-1.03, 0.76)	-0.30 (145)	0.768

BMI, body mass index; PSQI, Pittsburgh Sleep Quality Index (PSQI); N, number of subject; SD, standard deviation; CI, confidence interval; df, degree of freedom

* Methadone concentration at 24 h post-dose.

a *p* values were obtained using an unpaired independent t-test

**Table 2:** Comparison of cold pressor pain responses between patients with methadone concentration (at 24 h) of < 400 ng/mL and ≥ 400 ng/mL.

	**N**	**Adj. mean ** a	**95% CI**		***F *** **stat. (** ***df*** **)**	***p*** ** value ** b
			**Lower limit**	**Upper limit**		
Pain threshold (seconds)						
< 400 ng/mL *	87	30.15	24.29	36.01	5.59 (1)	0.019
≥ 400 ng/mL *	59	18.93	11.77	26.08		
Pain tolerance (s)						
< 400 ng/mL *	87	36.44	28.22	44.66	0.71 (1)	0.400
≥ 400 ng/mL*	59	30.82	20.79	40.86		
Pain intensity score						
< 400 ng/mL *	87	63.75	60.56	66.93	2.51 (1)	0.115
≥ 400 ng/mL *	59	67.84	63.94	71.73		

N, number of subject/allele; CI, confidence interval

* Methadone concentration at 24 hours post-dose.

a Adjusted mean controlling for daily methadone dose

b *p* values were obtained using repeated measure analysis of variance (RM-ANOVA) with covariates (*p* value is significant at < 0.05)

**Figure 1:**
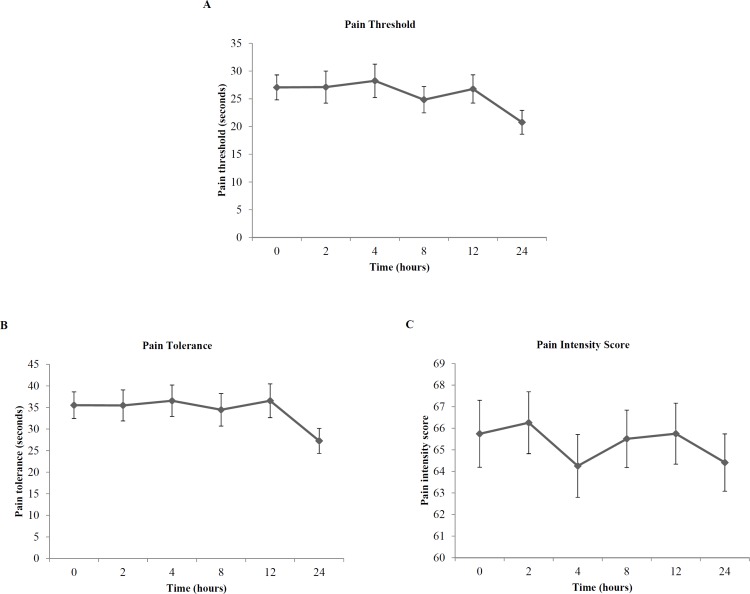
Profile Plots of Mean (SE) Pain Responses in the Opioid Dependent Patients. (A) Cold Pressor Pain Threshold. (B) Cold Pressor Pain Tolerance. (C) Cold Pressor Pain Intensity Score

**Figure 2:**
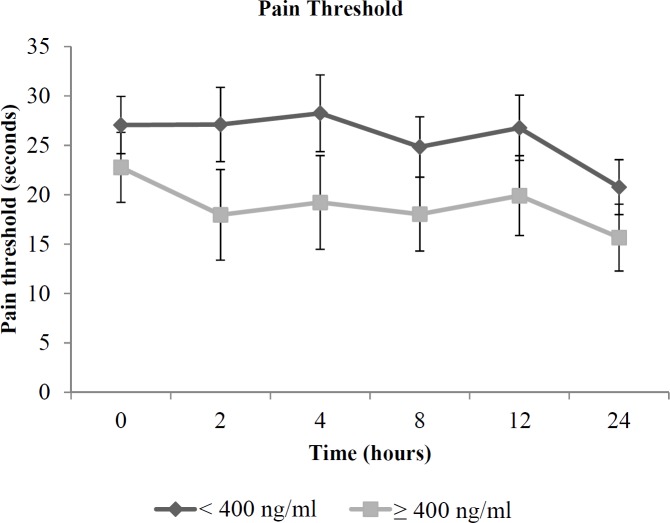
Profile plot of mean (SE) cold pressor pain threshold in the opioid dependent patients with serum methadone concentration (SMC) at 24 h of < 400 ng/mL and ≥ 400 ng/mL

Clotted blood samples taken from patients receiving MMT were centrifuged at 3,000 rpm for 10 minutes using the Thermo Scientific™ Sorvall™ ST 16 Centrifuge (Thermo Fisher Scientific Inc., Osterode, Germany) to separate the cells. The resulting supernatant (liquid component or serum) was immediately transferred into a clean 2 mL screw cap polypropylene tube. The serum was apportioned into 1.0 mL aliquots and stored at – 20 °C until use. Multiple freeze-thaw cycles of the serum samples was avoided to minimize protein degradation*.*

The serum methadone concentrations (SMCs) were measured using the three-step ELISA method, Methadone ELISA Kit that was developed and validated by other researchers in the Pharmacogenetics and Novel Therapeutics Cluster, Institute for Research in Molecular Medicine (INFORMM) as previously described (30, 34). The method proved to have good sensitivity, with a limit of quantification (LOQ) of 50 ng/mL (30,34). 

The Methadone ELISA Kit uses a competitive enzyme immunoassay method to determine the amount of methadone present in a serum sample and it is suitable for routine use in clinical settings (35). This kit allows 96 analyses (including the calibrators) or up to 40 samples in duplicate to be carried out per 96-well microtiter plate. The kit was stored at 2 – 8 °C and was brought to room temperature (20 – 25 °C) before use (35). 

*Statistical analysis*

Continuous variables were summarized as mean (SD) and categorical variables as frequency. The serum methadone concentration was classified into two categories: < 400 ng/mL and ≥ 400 ng/mL. The effects of serum methadone concentration were analyzed by comparing the cold pressor pain responses in opioid dependent patients with methadone concentration at 24 h post-dose of below 400 ng/mL and 400 ng/mL and above by means of repeated measures analysis of covariance (RM-ANCOVA), adjusting for methadone dosage. The limit of significance was set at 0.05. The statistical analysis was carried out using SPSS/Win software (Version 22, SPSS, Inc., 

Chicago, IL). 

## Results

*Patients*

Results were based on the data of 147 participants. The mean age was 36.89 years (SD = 6.14, range: 25 – 55 years). Subjects had been undergoing MMT for a mean of 2.83 years (SD = 2.02, range: 0.33 – 9.00 years). All MMT subjects were in the stable maintenance phase of treatment, the dosage of which had been individually optimized and had not changed for more than one month; this allowed determinations of the trough steady-state methadone concentrations. Out of the 169 potential subjects, 148 took methadone in a single dose (usually in the morning) and six split their daily dose into two portions. One patient had incomplete blood samples at 24 h. The other 15 patients were excluded for miscellaneous reasons. The mean daily methadone dose was 72.70 mg/day (SD = 28.25, range: 20 – 160 mg/day). Ninety-three patients (63.3%) received a daily methadone dose of < 80 mg/day and 54 (36.7%) received daily methadone dose of ≥ 80 mg/day. 

*Cold pressor pain responses in opioid dependent patients*

Figure 1 shows the mean cold pressor pain responses in opioid dependent patients. Incomplete cold pressor pain responses occurred in one patient at 24 h. At 24 h, cold pressor pain threshold ranged from 3.85 to 300 s, with a 78-fold variation. Cold pressor pain tolerance ranged from 6.88 to 300 s and cold pressor pain intensity scores ranged from 10 to 100, with a 44-fold and 10-fold variation, respectively.

*Serum methadone concentration (SMC) data*

The mean (SD) methadone concentration at 24 h (in ng/mL) was 387.78 (309.42) and it ranged from zero to 1672 ng/mL. Eight patients had trough methadone concentrations below the lower limit of quantification of 50 ng/mL at 24 h (their methadone concentrations were recorded as zero). When we applied a lower limit of 95% confidence interval for mean of 337.35 ng/mL and an upper limit of 438.22 ng/mL of trough methadone concentration on our sample, we detected that 78 (53.1%) patients had concentration below the lower limit and 52 (35.4%) above the upper limit. Eighty-eight patients (59.9%) had concentrations of below 400 ng/mL and 40.1% had 400 ng/mL and above, 400 ng/mL being the proposed necessary concentration to provide stabilized maintenance (31-33). 

The comparisons between patients with concentrations of below 400 ng/mL and those with concentrations of equal to or more than 400 ng/mL in demographic characteristics are shown in Table 1. The two groups were well matched with respect to age, weight and body mass index (BMI) (*p* > 0.05). There was a difference between groups for methadone dose (*p* = 0.003), so that patients with concentrations of below 400 ng/mL received significantly lower daily methadone dose than those with concentrations of 400 ng/mL and above.

In addition, separate analysis of the methadone dosage revealed that significantly higher methadone concentrations were observed in patients treated with methadone at dosages of ≥ 80 mg/day [mean (SD) = 506.62 (337.48) ng/mL] compared with those treated at lower dosages of < 80 mg/day [mean (SD) = 318.78 (270.60) ng/mL] (*p *< 0.001). 

*Influence of serum methadone concentration (SMC) on cold pressor pain responses*

The results of repeated measures ANCOVA, after controlling for daily methadone dose revealed significant effects of serum methadone concentration on cold pressor pain threshold (*p* = 0.019), but not on cold pressor pain tolerance and cold pressor pain intensity score (*p* > 0.05) (Table 2). 

The cold pressor pain threshold in opioid dependent patients, according to serum methadone concentration groups (concentrations of < 400 ng/mL and ≥ 400 ng/mL), adjusted for daily methadone dose are shown in Figure 2.

Patients having methadone concentrations of < 400 ng/mL had significantly higher cold pressor pain threshold (adjusted mean (95% CI) = 30.15 (24.29, 36.01) s) compared to those with concentrations of ≥ 400 ng/mL (18.93 (11.77, 26.08) s). In addition, exclusion of data from eight patients who had concentrations below the lower limit of quantification of 50 ng/mL did not alter this association (30.18 s and 19.01 s, respectively, *p* = 0.025).

## Discussion

Earlier studies reported large inter-individual variability in the maintenance dose of methadone and serum methadone levels for therapeutic response (36, 37). Similar observations were also observed in our current study. There was a wide variation in the individual daily doses of methadone prescribed for patients in our methadone maintenance programme (8-fold variation). 

The detected trough methadone concentrations also showed a wide inter-individual variability. In methadone maintenance therapy, methadone concentrations of 400 ng/mL are considered as necessary concentration to suppress any further opiate action and to provide stabilized maintenance (31-33). We found that the majority of our patients stabilized on the MMT had trough concentrations below 400 ng/mL. There were eight patients who were stabilized on very low methadone levels in blood (concentrations below the lower limit of quantification of 50 ng/mL). On the other hand, there were 14.3% patients who had potentially toxic concentrations of more than 700 ng/mL (34). These findings suggested that patients stabilized on the MMT may need unusually high or low methadone levels in plasma. 

The cold pressor test has confirmed a high inter-individual variability in the cold pressor pain responses. These findings suggested that wide inter-individual dose and concentration variations may contribute to a high inter-individual variability in the response to methadone treatment. 

The results of our study showed that patients with concentrations of < 400 ng/mL had an average 59.3% higher cold pressor pain threshold significantly different compared to those with concentrations of ≥ 400 ng/mL. It should be noted that exclusion of data from eight patients who had concentrations below the lower limit of quantification of 50 ng/mL did not alter this association; therefore it is unlikely that the data are biased by the inclusion of these subjects in the overall analysis. To the best of our knowledge, data on the relationship of methadone concentrations in blood with the corresponding pain sensitivity among opioid dependent patients is not available for reference.

The mechanisms behind our observations are unclear; however, it might be possible that patients with higher trough serum methadone concentrations may result in higher or more sustained levels of methadone at the site of its action in the brain, thus potentiating more hyperalgesia and subsequently, increased sensitivity to cold pressor pain. However, we did not obtain data on methadone brain levels or methadone central nervous system (CNS) distributions in our current study.

## Conclusion

In summary, this study reports a possible relationship between trough serum methadone concentration and cold pressor pain threshold. As such, it indicates that identifying the trough serum methadone concentration of opioid dependent patients on MMT may allow individualization of methadone substitution treatment. Results of serum methadone concentrations would be of particular importance during management of pain in this patient population, when it is critical to avoid misconception such as “drug-seeking” behaviors and more importantly, treatment failures such as patient relapsing to active substance abuse. In the future, doctors or other health care providers working in maintenance programs may apply the results of pain sensitivity test and methadone concentration to provide pain relief for opioid dependent patients who are in need of pain management in order to improve patient care and overall treatment outcomes.
